# Confocal Laser Endomicroscopy for Diagnosis and Histomorphologic Imaging of Brain Tumors *In Vivo*

**DOI:** 10.1371/journal.pone.0041760

**Published:** 2012-07-24

**Authors:** Sebastian Foersch, Axel Heimann, Ali Ayyad, Gilles A. Spoden, Luise Florin, Konstantin Mpoukouvalas, Ralf Kiesslich, Oliver Kempski, Martin Goetz, Patra Charalampaki

**Affiliations:** 1 Institute of Neurosurgical Pathophysiology, University Medical Center, Mainz, Germany; 2 Medical Clinic I, University Medical Center, Mainz, Germany; 3 Department of Neurosurgery, University Medical Center, Mainz, Germany; 4 Neurosurgical Department, Medical University Graz, Graz, Austria; 5 Department of Medical Microbiology and Hygiene, University Medical Center, Mainz, Germany; 6 Institute of Microtechnology Mainz, Mainz, Germany; The Ohio State University Medical Center, United States of America

## Abstract

Early detection and evaluation of brain tumors during surgery is crucial for accurate resection. Currently cryosections during surgery are regularly performed. Confocal laser endomicroscopy (CLE) is a novel technique permitting *in vivo* histologic imaging with miniaturized endoscopic probes at excellent resolution. Aim of the current study was to evaluate CLE for *in vivo* diagnosis in different types and models of intracranial neoplasia. *In vivo* histomorphology of healthy brains and two different C6 glioma cell line allografts was evaluated in rats. One cell line expressed EYFP, the other cell line was used for staining with fluorescent dyes (fluorescein, acriflavine, FITC-dextran and Indocyanine green). To evaluate future application in patients, fresh surgical resection specimen of human intracranial tumors (n = 15) were examined (glioblastoma multiforme, meningioma, craniopharyngioma, acoustic neurinoma, brain metastasis, medulloblastoma, epidermoid tumor). Healthy brain tissue adjacent to the samples served as control. CLE yielded high-quality histomorphology of normal brain tissue and tumors. Different fluorescent agents revealed distinct aspects of tissue and cell structure (nuclear pattern, axonal pathways, hemorrhages). CLE discrimination of neoplastic from healthy brain tissue was easy to perform based on tissue and cellular architecture and resemblance with histopathology was excellent. Confocal laser endomicroscopy allows immediate *in vivo* imaging of normal and neoplastic brain tissue at high resolution. The technology might be transferred to scientific and clinical application in neurosurgery and neuropathology. It may become helpful to screen for tumor free margins and to improve the surgical resection of malignant brain tumors, and opens the door to *in vivo* molecular imaging of tumors and other neurologic disorders.

## Introduction

Intracranial neoplasms include a variety of different histopathologic entities, ranging from rather benign tumors, such as meningiomas, to some of the most aggressive types of human cancer. Glioblastoma multiforme, for example, is the most frequent primary malignant brain tumor in adults and median survival is on average less than one year from the time of diagnosis [1]. Even in the most favorable situations, the majority of patients has a median survival of 18–21 months [2]. Studies have shown that - as in almost every other tumor disease - early and precise diagnosis [3] and immediate multi-modal treatment is crucial for improving survival rates and quality of life in patients with brain tumors [2], [4]. Another key factor with patients undergoing neurosurgical intervention due to intracranial neoplasia is the total removal of the tumor [5], while at the same time minimizing trauma to healthy brain tissue. Indeed, in a review of every major clinical publication since 1990 on the role of extent of resection in glioma outcome Sanai and Berger [6] concluded that despite persistent limitations in the quality of data, mounting evidence suggests that more extensive surgical resection is associated with longer life expectancy for both low- and high-grade gliomas. Similarly, Stummer et al. [2] compared three recent randomized phase 3 trials and suggest that complete resection should be the surgical goal for glioblastoma. This is crucial, since increased surgical radicality naturally bears a higher risk of damage to important intact cerebral regions and pathways. Although neurosurgeons have recently been equipped with new technologic features, such as neuro-navigation [7] and fluorescence-guided surgery [8], distinction of healthy and tumor cells on a cellular level remains challenging.

Optical technologies have continuously contributed to the advancement of diagnostics and therapeutics in modern medicine. A current example is a novel technique that has been introduced recently to gastroenterology: confocal laser endomicroscopy (CLE). New miniaturized scanners, integrated into the tip of normal endoscopes, allow immediate microscopy of different tissues with high-resolution *in vivo* histologic visualization of cellular, subcellular and even subnuclear structures during ongoing endoscopy or laparoscopy [9]. In-vivo histology is now possible at lateral and axial resolution <0.1 µm with nearly 1000-fold magnification [10]. Tissue contrast and staining can be achieved by administration of various fluorescent dyes, which can be used to elucidate different morphological and also functional histologic aspects [11]. In combination with antibodies and other targeting molecules, endomicroscopy can even be used for molecular targeted imaging *in vivo* [12]–[14]. CLE imaging of neurosurgical disorders, such as intracranial neoplasia, has only recently been suggested and miniaturized CLE probes could possibly contribute to both immediate tumor detection and inspection of resection margins during ongoing surgical procedure[15]–[17]. Endomicroscopy provides the physician with the direct histomorphological aspect of the examined tissue and was used, for example, in the gastrointestinal tract to precisely characterize and distinguish between a wide variety of different types of healthy and altered tissue[18]–[21]. With its small-sized optics it might also be suited for endoscopic and minimally invasive surgery in ENT and urologic oncologic procedures [22], [23].

In an initial trial, we demonstrated imaging of microvasculature and perfusion using CLE [24]. Aim of the current study was 1) to evaluate in general a potential use of CLE for neurosurgical applications, 2) the role the technique could play for in vivo real time histology, and lastly 3) to show potential advances for distinguishing intraoperatively normal from tumor tissue, which could help to improve surgical resection of malignant brain tumors. We first examined different C6 glioma cell line allografts implanted into the brain of healthy Wistar-rats *in vivo* using CLE. This was (A) to show general feasibility by using allografts expressing endogenous fluorescence without any dyes and (B) to investigate a variety of different fluorescent agents in wildtype C6-glioma allografts. Emphasis was put on the distinction between healthy and altered tissue, and on the identification of the borders. To evaluate further clinical application for real time intraoperative histology, and to develop a set of endomicroscopic criteria, we used fresh resection specimens of different types of brain tumors for direct confocal endomicroscopic imaging *ex vivo*. Conventional histopathology served as gold standard in all models and representative images were used for statistical evaluation by blinded raters.

## Materials and Methods

### Ethical Considerations

Animal procedures were in accordance with national and international guidelines and were approved by the governmental animal care and use committee (Landesuntersuchungsamt Koblenz 23-177-07/G 10-1-050). According to the contract existing between every admitted patient (including written informed consent) and the University Medical Center Mainz, Germany, tumor biopsy material could be used for medical research if that material was not needed for other purposes. This contract was composed following strict recommendations given by the ethics committee of the Rhineland-Palatinate medical association and approved by the board of governors of the University Medical Center Mainz.

### Confocal Laser Endomicroscopy

**Figure 1:**
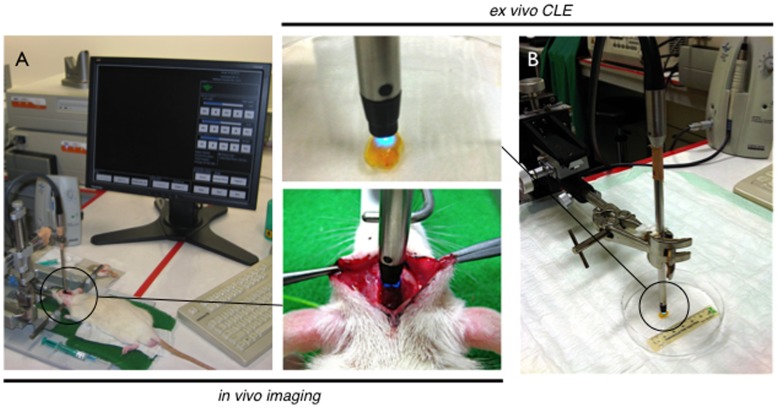
Experimental setting. A - In vivo confocal laser endomicroscopy in anesthetized, tumor-bearing wistar rats. The animal is positioned in a stereotactic frame, which is connected to the confocal endomicroscopic probe. Detail: the tip of the probe is gently brought in contact with the intracranial window and images of the tumor, the transition zone and healthy brain tissue are gathered. B - Ex vivo imaging of fresh surgical resection specimen of brain tumors using CLE. Samples were gathered intraoperatively and immediately transferred to the imaging facility. Detail: imaging after topical staining with acriflavine.

The confocal laser endomicroscopy probe (CLE) (Optiscan Pty Ltd., Notting Hill, Victoria, Australia) uses a single optical fiber for both the excitation source and the detection pinhole. The probe is ﬂexibly connected to the laser source and the detection and image processing CPU. The solid-state blue laser uses an excitation wavelength of 488 nm - light emission was detected at 505–585 nm. The laser-beam is navigated by an electromagnetically actuated control system offering resonant scanning in both x- and y-axis and is directed into the specimen via a miniature multielement objective lens. Lateral resolution is close to 0.7 µm. Z-axis actuation is achieved by an electrically controlled shape-memory-alloy, which enables the scanning of the tissue as deep as 250 µm from the imaging window in 4 µm increments. Vertical resolution (“optical slice thickness”) is approximately 7 µm. Each raster-scanned image is a horizontal optical section of 500×500 µm in size. The miniaturization allows an integration of the CLE imaging head into a handheld rigid probe **(** **Figure 1** **, Fig S1)** with an outer diameter of 7 mm - making the probe well suited for endoscopic or surgical procedures, especially in neurosurgical applications. The rigid probe is commercially available in different lengths. Identically constructed probes have been integrated into standard gastroenterologic endoscopes, and are well established in clinical practice. The probe can be used as a handheld device or mounted onto a micromanipulator to minimize motion artifacts. Serial images are collected at a scan rate of 0.8 frames per second at a resolution of 1024×1024 pixels or 1.6 frames per second at 1024×512 pixels, approximating a 1000× magnification on a 19-inch screen. Details can be further magnified digitally using image software (up to 100000×) by changing into a review mode during the ongoing examination.

### Fluorescent Agents

Different ﬂuorescent agents were used for tissue contrast and highlighting of certain morphological features. In the *in vivo* allograft experiments, Na^+^ fluorescein (Alcon Pharma, Freiburg, Germany) was injected intravenously at 100 µg/g bw. Depending on incubation time it could be used for displaying different intercellular tissue features. Acriﬂavine (Sigma Pharmaceuticals, Victoria, Australia) was administered either topically or intravenously. For topical staining, several drops of a 0.01% solution in PBS were applied to the tissue surface, and excess dye was washed away with saline. For systemic application, 10 µg/g BW acriﬂavine was injected. Acriﬂavine integrates into the dsDNA and is used for staining interphase nuclei and mitotic cells. Indocyanine green was used intravenously at 100 µg/g BW, for vessel and general morphology (data not shown). Fluorescein isothiocyanate-(FITC-)labeled dextran (MW 150 kDa; Sigma-Aldrich, Steinheim, Germany) was injected intravenously at a dose of 250 µg/g bw. FITC-dextran is a high-molecular sugar to visualize blood ﬂow and vessel leakiness. The concentration and volume of ﬂuorescent agents used and the incubation times for each agent were optimized by immediate evaluation of the collected images. A mid-laser power of 300–600 mW was used on the CLE probe to record stainings. Human tissue samples were only incubated with acriflavine.

### Cell Culture and Allograft Implantation

C6 glioma cells were obtained from the Institute of Neurosurgical Pathophysiology, Mainz, Germany and cultivated in Dulbecco’s modified Eagle medium (DMEM) supplemented with 1% penicillin, 1% streptomycin, 10% fetal calf serum at 37°C and 5% CO_2_ atmosphere. C6 cell lines expressing the autofluorescent green ﬂuorescent protein mutant, EYFP, were generated by cotransfection of C6 wild type cells (C6^WT^) with vectors pEYFP-C1 (Clontech) and pBabePuro (Addgene, Cambridge, USA). Transfection was performed by using Lipofectamine (Invitrogen, Karlsruhe, Germany) and cells were selected by addition of puromycin (2 µg/ml; Sigma Aldrich, Munich, Germany) for 21 days. Stable expression of EYFP in the selected cell line (C6^EYFP^) was controlled in living cells by ﬂuorescence microscopy using the appropriate excitation and emission filters (IX70, Olympus, Hamburg, Germany) as described [25]. Cells were harvested routinely after 5–10 days using Trypsin-EDTA (0.05%, 0.02% resp.) and suspended in saline.

### Animals and Allograft Tumors

Prior to injection, viability of the used C6^WT^ or C6^EYFP^ tumor cells was assessed by trypan blue staining. C6^WT^ or C6^EYFP^ glioma cells (1×10^6^ - 8/3 animals/group) were implanted stereotactically into the left frontal region of the brain of anesthetized young adult male Wistar rats (Charles River Wiga, Sulzfeld, Germany) as described [26]. Briefly, burr-hole trephination was carried out and a custom-designed needle was inserted for slow injection of the tumor cell suspension. Tumors were allowed to establish for ten days on average and animals were then used for *in vivo* confocal imaging. After this time period, animals implanted with both C6^WT^ or C6^GFP^ glioma cells begin to show signs of apathy and fatigue. Animals were bred and kept at the animal facility in a temperature-controlled environment on a 12- hour light/dark cycle and were fed a regular pelleted rodent maintenance diet and water *ad libitum*.

### *In vivo* Confocal Imaging of Allograft Tumors

An intraperitoneal catheter was placed for deep anesthesia with chloral hydrate and intravenous catheters were inserted for drug application. Craniotomy was performed in a stereotactic frame and an intracranial window was placed to expose the allograft tumor, the transition zone and healthy brain tissue, while bleeding was controlled using heat-coagulation, TABOTAMP® and bone wax (Henry Schein VET, Hamburg, Deutschland). For *in vivo* imaging the probe was placed directly onto the tissue by using it as a handheld device or by mounting it in the stereotactic frame. The macroscopically visible C6 glioma allograft tumors were scanned with the rigid probe and special attention was paid to the transition zone between the neoplastic and normal brain tissue. Multiple tumor and healthy brain tissue sites were scanned and focal lesions were identified. A total of 50 to 250 grayscale images were collected per examination. After *in vivo* imaging, the rats were perfusion-fixed with 4% buffered paraformaldehyde and the brain was removed for further histopathologic evaluation. Tissue specimens were taken directly from the site of the endomicroscopic examination for correlation.

### Collection and Confocal Imaging of Human Tissue

Biopsy specimens of patients diagnosed with intracranial neoplasia and treated at the Department of Neurosurgery of the University Medical Center Mainz were analyzed. Studies of human tissue biopsies were performed according to the requirements of the local ethics committee, and in accordance with the Declaration of Helsinki. Tumor specimens and healthy brain tissue were collected from patients who underwent surgery. All cases were histopathologically diagnosed according to established criteria by routine pathology. Fresh surgical samples (3–10 mm) were immediately placed in PBS at 4°C and incubated in the dark with 50 µl acriﬂavine or fluorescein for 3–5 min. at room temperature. Excessive label was removed by three washing steps with PBS and specimens were immediately examined by CLE using the probe hand-held or held by a micromanipulator. Subsequently, the specimens were transferred to 4% buffered formalin or frozen in liquid nitrogen for histopathology (Table 1).

**Table 1:** Origin and number of human tumor samples studied by confocal laser endomicroscopy.

Number of samples	Routine histopathogy diagnosis
6	Meningioma
1	Glioblastoma
2	Acoustic neurinoma
1	Epidermoid tumor
1	Craniopharyngioma
1	Brain metastasis of a primary mamma carcinoma
1	Medulloblastoma
2	Healthy brain tissue (adjacent to tumor tissue)

**Figure 2:**
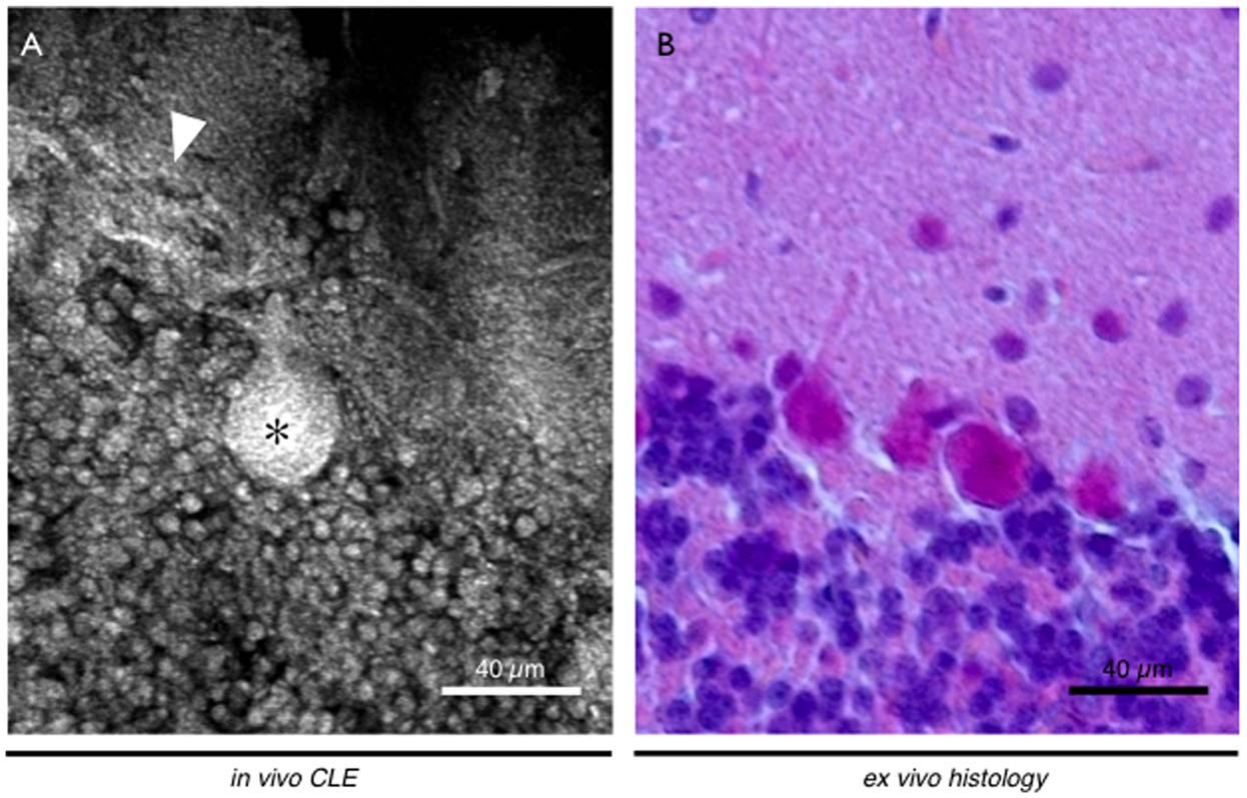
Healthy cerebellum of a Wistar rat. A - *In vivo* CLE enabled to image the histology of different parts of the brain, e. g. the cerebellum. After topical application of acriflavine, the characteristic layers of can be observed and specific Purkinje cells (asterisk) with their dendritic tree (triangle) are stained. B - The resemblance to ex vivo histopathologic staining is substantial.

**Figure 3:**
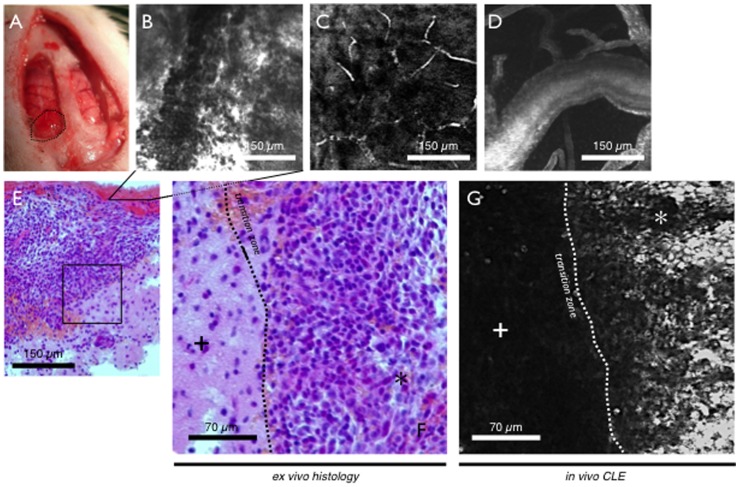
C6^WT^ glioma allograft. A - Macroscopic view of two intracranial windows. On the left side the transition zone between healthy and tumor tissue can be observed. The other side served as control. B-D - Vessel morphology after intravenous injection of FITC-dextrane. (B) Tumor tissue shows massive extravasation with erythrocytes and FITC-dextrane in the interstitial zone (also seen in H&E staining). (C & E) Normal vessels and capillaries reveal structural integrity and erythrocytes can be observed in their physiological flow. E-G - Transition zone between healthy brain tissue (cross) and C6^WT^ glioma allograft (asterisk) displayed *in vivo* using CLE (G) and ex vivo with H & E staining (E, F). Both entities show their specific histomorphological aspect in either one procedure.

**Table 2:** Comparison of confocal endomicroscopy with conventional histopathology.

Species	Entity	histopathology	Endomicroscopy
Rodent	Cerebellum	*H&E:* characteristic layers, purkinjecell soma.	*Fluorescein: c*haracteristic layers, purkinje cell soma and dendritic trees.
	Cerebrum	*H&E:* neurons, glial cells *Nissl-staining:* neurons and fibers.	*Fluorescein: c*apillaries, neurons, fibers, glial cells. *FITC-dextrane: v*essels (arterioles and capillaries). *Acriflavine:* Nuclei of neurons and glial cells. *Indocyanine green:* vessels (arterioles and capillaries).
	C6^wt^ glioma	*H&E:* rampant tumor growth, atypiccells, shift in nuclear-to-cytoplasmratio, mitoses.	*Fluorescein:* rampant tumor growth, massive extravasation*FITC-dextrane:* atypic tumor vessels, massive extravasation.*Acriflavine:* rampant tumor growth, atypic cells, shift in nuclear-to-cytoplasm ratio, mitoses.
Human*(only H&E,* *acriflavine)*	healthy brain	Neurons, glial cells.	Nuclei of neurons and glial cells
	Glioblastoma	Rampant tumor growth, atypic cells,shift in nuclear-to-cytoplasm ratio,mitoses.	Rampant tumor growth, atypic cells, shift in nuclear-to-cytoplasm ratio, mitoses.
	Meningioma	Benign nuclear morphology,psammoma bodies.	Benign nuclear morphology, psammoma bodies.
	Acoustic neurinoma	Antoni A and B fibers, Verocay bodies.	Antoni A and B fibers, Verocay bodies.
	Craniopharyngioma	turbulent small nuclear pattern, squamous origin.	Turbulent small nuclear pattern (no signs of higher malignacy), squamous origin.
	Brain metastasis	large malignant tumor cells,mitoses, atypic nuclei.	Large malignant tumor cells, mitoses, atypic nuclei.
	Epidermoid tumor	cell fragments.	Pentagonal cells, mosaic pattern, epithelial origin

### Histopathology

Human or allograft tissue samples were formalin fixed, paraffin embedded (FFPE) and processed for histopathology. 3–5 µm sections were placed on object slides for histopathological staining. To evaluate correlation and morphological resemblance with *in vivo* endomicroscopic images, haematoxilin and eosin staining was performed according to standard protocols [12] and sections were examined with a conventional white light and fluorescence bench top microscope (IX70, Olympus, Hamburg, Germany). For imaging of neuron fibers and axons, Cresyl violet (Nissl-) staining was used. Ex vivo bench top fluorescence microscopy was used to confirm the imaging results achieved with confocal laser endomicroscopy. A conventional fluorescence microscope (IX70, Olympus, Hamburg, Germany) was used after cryo-sectioning the tissue samples. Since EYFP-positive cells provided a specific fluorescent signal no further staining procedure was carried out, except nuclear counterstaining with DAPI (Vector Laboratories, Burlingame, USA).

### Statistical Analysis

To establish accuracy and interobserver agreement of confocal endomicroscopy, a set of confocal images (n = 35) of 7 different tissues were selected and presented to two groups of raters: non-clinical experts (GS, LF) and clinical experts (OK, PC, AH) who were blinded to the macroscopic appearance and the histopathological diagnosis of routine pathology. The relationship between qualitative variables and the comparison of relative numbers were examined by using contingency tables.

**Figure 4:**
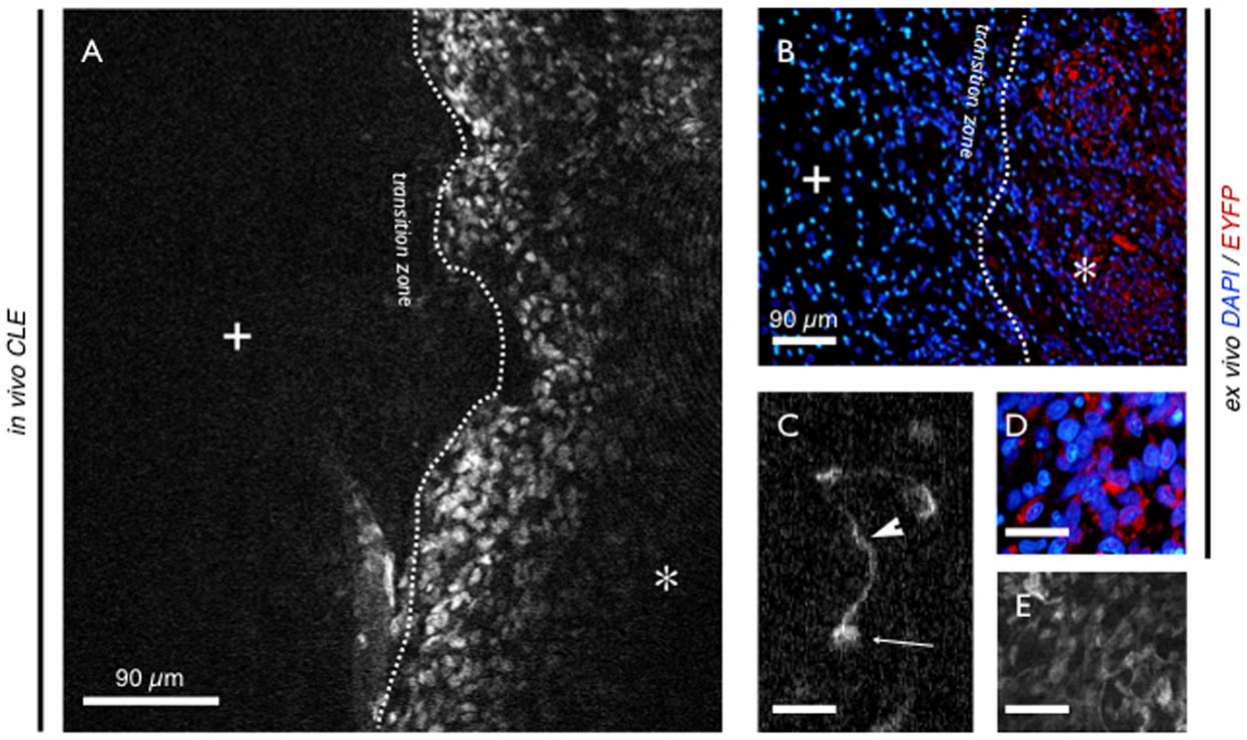
C6^EYFP^ glioma allograft. A - Image of the transition zone between healthy brain tissue (cross) and tumor tissue (asterisk) with endogenous fluorescence *in vivo* using CLE. No added fluorescence was used for intravital imaging. Where allograft tissue reveals a characteristic histomorphology, healthy brain shows no signal at all. B - Ex vivo bench top fluorescence microscopy with DAPI nuclei counterstaining. Again the transition zone can be observed. C - CLE shows cellular and subcellular details of the tumor cells: as cells of glia-origin appendices (triangle) and cell soma (arrow) can be distinguished. D, E - Detail of fluorescent tumor cells in ex vivo bench top fluorescence and *in vivo* CLE. High resemblance between both imaging modalities was found. - Scale bars c – e: 30 µm.

**Figure 5:**
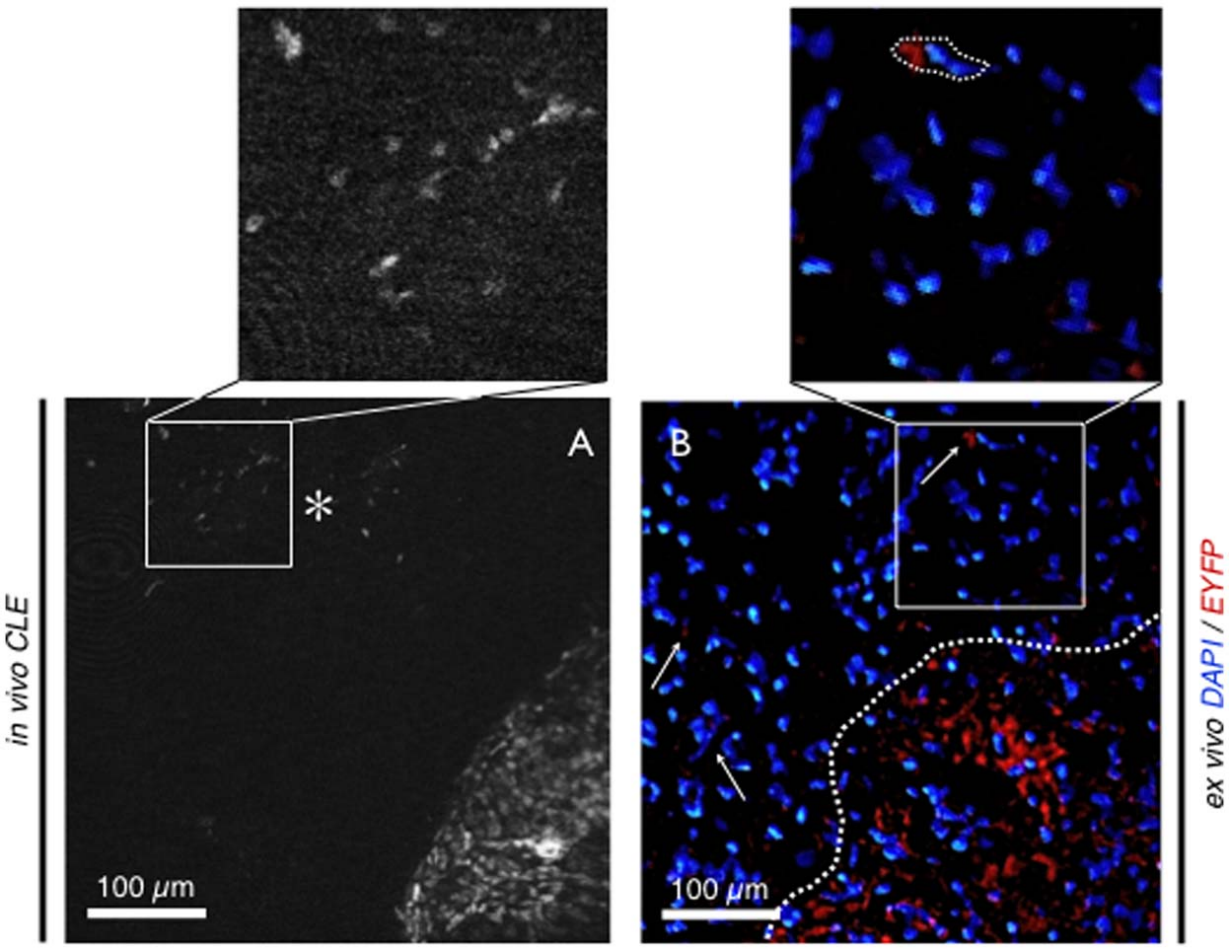
C6^EYFP^ glioma allograft. Invading tumor cells distant from the main tumor (asterisk) can be observed in vivo (A) and are confirmed by ex vivo fluorescence microscopy (B). No fluorescent dye was applied for imaging in vivo. Since no auto-fluorescence was observed, specific signal results from transfected tumor cells. Ex vivo, no further staining procedure was performed, except counter staining of the nuclei with DAPI. Fluorescent tumor cells are also seen distant from the main tumor (arrows).

## Results

### *In vivo* Imaging of Brain Tumor Allografts

In n = 11 allograft bearing rats *in vivo* confocal endomicroscopic imaging was performed (Table 2). In n = 3 animals, intravenous application of fluorescein resulted in a strong staining to observe overall brain and tumor morphology. First, the structure of small vessels and capillaries could be seen, after several minutes fluorescein diffused into both healthy brain and tumor tissue. Tissue staining could be observed faster and more intensively in malignant tissue compared to normal brain tissue due to capillary leakage via the incomplete blood-brain-barrier (BBB). Depending on the location of the confocal imaging window, healthy brain tissue showed a low number of prominent triangular cells, likely mimicking neurons. Smaller cells – potentially of glial origin - could also be observed and axon pathways, as well as medullar structures could be seen in deeper areas of the brain. CLE also enabled *in vivo* imaging of the cerebellum, where characteristic stratification into molecular layer, Purkinje layer, nuclear layer and medullar layer could be observed. Especially Purkinje cells could be seen clearly with their dendritic branches, large cell somas and tree like shapes (Figure 2). In tumor tissue strong fluorescence depicted massive extravasation and cell rich stroma could be displayed. Small intratumoral hemorrhages are represented by erythrocytes flowing into the interstitial space (Figure 3).

Acriflavine was evaluated for a nuclear staining pattern in n = 3 rats. In normal tissue scattered nuclei of healthy neurons could be observed with characteristic morphology. Due to the high resolution of the confocal probe, even subnuclear distribution of different types of chromatin could be examined *in vivo*. Singular prominent nucleoli were seen frequently as typical features of cells (microglia or neurons, resp.)-of the outer cortical layer. In tumor tissue, rampant cell growth and high density of irregularly shaped glioma cells were displayed. Typical signs of malignancy, such as shift of nuclear-to-cytoplasm ration and increased number of mitosis were found in vivo. The distinct histomorphological differences between healthy and neoplastic brain tissue could be observed particularly well at the transition zone. It was possible on a cellular level to visualize the tumor margin and differentiate between both entities *in vivo* (Figure 3).

Imaging of C6^EYPF^ allografts resulted in good to excellent visualization of EYFP exprespsing tumor cells without any further staining procedure in n = 5 rats. Characteristic histomorphology of the glioma cells could be displayed. The transition zone between healthy brain tissue and the tumor could easily be found as the area where the strong cellular fluorescent signal disrupted. CLE even showed microscopic details of the tumor cells with subcellular resolution. As tumor cells of glial origin, characteristic appendices and cell somata could be observed (Figure 4). Interestingly single tumor cells could be seen distant from the main tumor migrating and infiltrating into normal tissue (Figure 5), demonstrating deeper infiltration of healthy brain tissue.

After FITC-dextran (and to a lesser extent indocyanine green) injection, vessels such as small capillaries and also larger supplying vessels could be visualized in both healthy and allograft tissue in n = 2 rats. But whereas healthy brain vessels showed structural integrity and coordinated blood flow, tumor vessels were shaped irregularly and frequently showed massive leakage and extravasation of the FITC-dextrane, again due to the disturbed BBB (Figure 3). It was difficult to distinguish between different types of vessels and other intratumoral blood pathways due to high fluorescence intensity from intratumoral hemorrhages.

### Human Tissue

**Figure 6:**
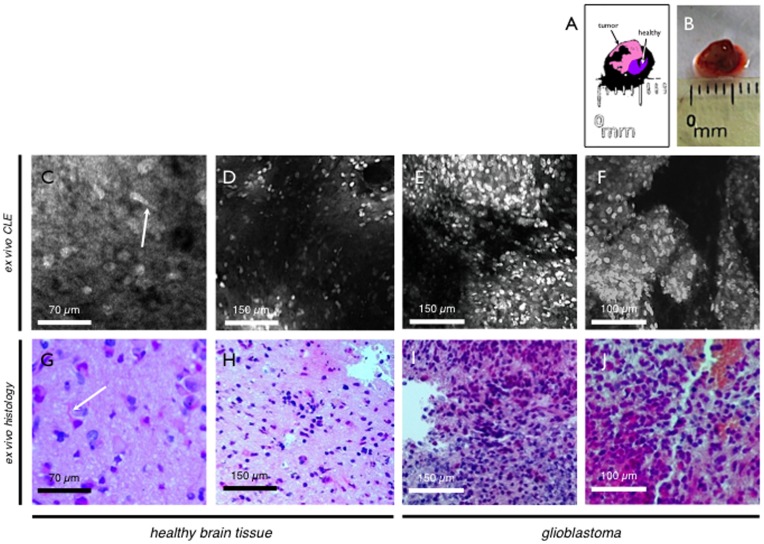
Glioblastoma. Schematic (A) and macroscopic (B) image of resection specimen. Attached to the tumorous tissue (pink), a small piece of healthy brain tissue was observed (purple). Panel C-F - *Ex vivo* CLE of a fresh surgical resection specimen of healthy brain tissue (C, D) in direct proximity of the glioblastoma specimen (E, F) of the same patient. Healthy brain tissue reveals neuron or microglial cells with appendices (arrows). In comparison to healthy brain tissue the tumor reveals rampant excessive growth with atypic nuclei and abnormal nuclear-to-cytoplasm ratio. Atypic mitoses can be observed. Panel G-J - Ex vivo histopathology confirms the findings and diagnosis found in CLE (healthy brain tissue G, H – glioblastoma I, J).

A total of n = 15 fresh biopsy specimens of different intracranial neoplasms and normal cerebral tissue were examined *ex vivo* with CLE. In all cases acriflavine provided satisfactory contrast for endomicroscopic imaging. Healthy brain tissue attached to a tumor specimen, showed typical cells with large nuclei mostly depicting neurons or cells of glial origin. Smaller interstitial cells were also observed, resembling stromal- or immune cells. Images showed typical characteristics of healthy rat brain tissue. (Figure 6). Human glioblastoma showed rampant and invasive cell growth. As in the C6 glioma allografts, common signs of malignancy could be observed such as nuclear polymorphisms, shift of nuclear-cytoplasm-ratio and high proliferation index, shown by atypical mitoses (Fig. 6).

**Figure 7:**
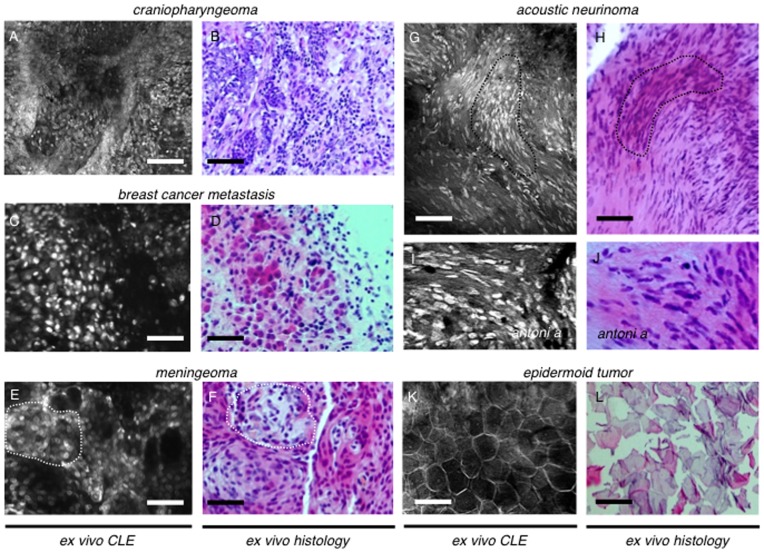
Fresh human brain tumor biopsy specimens. A - *Ex vivo* CLE of a craniopharyngioma. Small nuclei aligned in disarranged formations such as strands or trabecular structures represent the squamous origin of this mostly benign tumor. No calcifications can be observed using CLE or ex vivo (B) indicating the papillar type of this neoplasia. B - H.&E. staining of this lesion shows high similarity to CLE. C - Confocal images of a fresh resection specimen of a brain metastasis of a primary ductal mamma carcinoma. This tumor origin is consistent with the histomorphologic aspect of the neoplastic cells. Differences in size and shape of both the cells and the nuclei can be observed. Prominent nucleoli are also characteristic for the metastatic tissue. D - An excellent resemblance of the endomicroscopic images and the histopathologic gold standard can be observed. E, F - Typical example of a number of meningiomas, using CLE (E) and ex vivo (F). Characteristic pallisade-shaped and round cell formations can be observed representing psammoma bodies (outline). Tumor cell nuclei however remain mostly of equal size and shape, indicating the tumor’s benign origin. Again ex vivo histopathology by H.&E. staining strongly resembles the CLE images. G - Confocal laser endomicroscopy of an acoustic neurinoma. Nuclei accumulation can be frequently seen, consistent with the histopathologic feature of Verocay bodies (outline). Tumor fibers can be observed representing Antoni A structures (I). H, J - All characteristics of acoustic neurinoma can also be found in conventional ex vivo histopathology. K - An epidermoid cyst tumor was subject to the last confocal laser endomicroscopic examination. In CLE a crystal like structure could be observed with almost no nuclear staining. These cells represent desquamating epithelial cells. Massive keratin accumulation can also be observed. L - H.&E. staining again confirms findings of CLE. Scale bars represent 100 µm.

Craniopharyngioma showed a completely different histomorphologic aspect. Tumor cells could be observed forming round formations (Fig. 7 A,B, Fig. S2 A,B), which were, however, decisively different compared to the psammoma bodies seen in meningioma (Fig. 7 E,F). Nuclear morphology of craniopharyngioma tissue showed characteristic elliptic shapes, indicating the tumor origin from squamous tissue (Fig. 7 A,B, Fig. S2 A,B). A cell rich stroma could be observed in the biopsy specimen of brain metastasis with CLE *ex vivo*. Tumor cells showed a wide range in nuclear-to-cytoplasm ratio with the majority of the rather large cells being of round or oval shape. Many atypically shaped nuclei with prominent nucleoli and mitoses indicated malignant origin. Glandular layout was missing - consistent with ductal mammary carcinoma as the primary tumor (Fig. 7 C,D, Fig. S2 C,D). A total of 6 meningioma tumors were examined with CLE. Compared to healthy brain tissue these rather benign tumors showed a higher cellular density. But as opposed to malignant tumors such as glioblastoma, nuclear shape was more uniform and typically well-ordered. Characteristic features of meningiomas found *ex vivo* were round tumor cell formations depicting psammoma bodies and palisade-shaped tissue strands (Fig. 7 E,F, Fig. S3 A,B). CLE of acoustic neurinoma revealed the typical fibrous aspect of this tumor. The neoplastic cells, originating from Schwann cells of the vestibular nerve, formed characteristic fibers and cords. Clusters of atypically shaped nuclei most likely represented Verocay bodies. A nuclear staining pattern strongly resembling Antoni A fibers could be found in different parts of the specimen (Fig. 7 G-J). The epidermoid cyst also displayed a unique histomorphology *ex vivo* using CLE. Crystal like structures were a clear indication of the epidermal origin most likely representing desquamating epithelial cells. No nuclei could be found in the superficial cell layers (Figure 7 K,L; Fig. S3 C,D).

### Histopathology

H&E staining revealed the characteristic features of the different tumors. The resemblance between *ex vivo* CLE using acriflavine or fluorescein and ex vivo histopathologic staining was excellent. Especially histology from C6 glioma allografts and glioblastoma with healthy brain tissue attached suffered from cutting and staining artifacts which were not observed with *in vivo* CLE to that extent. Cresyl violet staining was performed for neuron fiber and axon pathway detection ex vivo. A strong resemblance was found between ex vivo cresyl violet staining and *vivo* CLE with fluorescein in sections of different parts of healthy brain (data not shown). Bench top fluorescence microscopy revealed a strong cellular signal without any further processing of the tissue. Again the resemblance to *in vivo* CLE was substantial. Infiltrating cells apart from the main tumor could also be observed using bench top fluorescence microscopy.

### Statistical Analysis

A total number of n = 175 representative endomicroscopic images of n = 7 different human tissues were evaluated by n = 5 different raters. All meningioma (30/30), glioblastoma (20/20), acoustic neurinoma (20/20) and epidermoid cyst tumors (25/25) images were diagnosed correctly. Craniopharyngioma tissue was recognized in 22/25 cases, healthy tissue in 25/30 cases and metastatic tissue in 17/25 cases. Overall correct detection rate was 90,85%. Two raters were declared as non-clinical experts with no direct connection to a neurosurgical or neuropathologic department. They scored a total of 87,14%, while clinical experts gave correct diagnosis in 93,33% of the cases.

## Discussion

In our pilot trial, we demonstrate that confocal laser endomicroscopy with new miniaturized probes allows histological imaging of healthy brain and tumors at microscopic level *in vivo* in rat allograft models and human tissue. The implementation of this technique in neurosurgery could pose a new and important application for clinical routine and research. By using different fluorescent agents we first successfully displayed the *in vivo* histomorphology of healthy rat brain tissue and C6 glioma allografts and were thus able to distinguish between both entities during ongoing examination. In fresh surgical resection specimens, *ex vivo* CLE could then be used for immediate real time and precise diagnosis of various intracranial neoplasia. To investigate if this novel imaging can be applied to human tissue, fresh tissue specimens of different human brain cancer were examined, providing a good signal intensity and adequate contrast for CLE imaging after topical application of acriflavine and fluorescein. Reproducible and specific histomorphologic criteria were found and resemblance with ex vivo histopathologic gold standard was substantial.

Until today, diagnosis of brain neoplasms heavily depends on neuroradiology and histopathology. But as CT or MRI can only provide a macroscopic view of a particular tumor and histopathology remains unclear, stereotactic biopsy still plays an important role in pre- and perioperative care in patients suffering from brain tumors - as Callovini et al. [27] described. Although it is relatively safe and effective to perform [28], conventional histology can only provide a snapshot of a small portion of the tumor and is subject to cutting-, embedding- and staining artifacts [29], [30]. Endomicroscopic equipment might easily be integrated into a minimally invasive or endoscopic setting and provides immediate and intraoperativ histopathologic diagnosis of the entire entity at real time which might be directly followed by therapeutic neurosurgical intervention.

A similar - but very promising - application would be the use of *in vivo* CLE during neurosurgical intervention, in particular during tumor resection. During neurosurgery for tumor resection cryosections are regularly performed by the neuropathologist. With CLE less cryosections may be required and tumor margins can even be determined by transmitting images online to the neuropathologist via an automated telepathological conference during surgery. This leads the surgeon performing more accurate and extended surgical resection identifying the tumor borders on a cellular level.

Even without CLE novel techniques that involve fluorescent agents have achieved significantly better surgical results as compared to conventional white light illumination [31], [32]. In combination with this technology, *in vivo* CLE could provide the valuable link to intravital microscopy to evaluate resection margins and minimize residual tumor volume. Minor drawbacks, such as sterilization of the confocal probe have already been solved [20], [33]. Based on the concept of “smart biopsies”, where the number of biopsies can be drastically minimized when combined with prior endomicroscopic procedure [34], this could possibly lead to a smaller number of rapid sections and biopsy specimens, reducing the risk of damage to healthy brain structures. Statistical evaluation of a representative set of endomicroscopic images has shown that even raters with no neurosurgical or neuropathological background can recognize a variety of different tissues correctly. The neuropathologist could also be on the spot in the operating room using telemedicine or video transmission.

There are limitations to our approach of *in vivo* histopathologic imaging using CLE. Intravenous injection or topical application of some fluorescent agents (Acriflavine and FITC-dextrane) are not approved to be used in a clinical neurosurgical setting. Therefore, we consider intravenous fluorescein and indocyanine green - both of which have been used in clinical practice for decades - more suitable up to now. But other fluorescent agents - which have not been examined in this study - are already available or subject to extensive research and can be used in humans [35]. Although we observed the strongest fluorescence signal on the tumor surface, the limited infiltration depth of the endomicroscope could be a possible drawback. However, this could be overcome with the next generation of confocal systems, for example near infrared (NIR) probes. NIR confocal imaging with indocyanine green has already been investigated for liver imaging during laparoscopy with a prototype probe [9]. In the future, even confocal systems with multiple excitation wavelengths such as in bench top confocal imaging may facilitate clinical use. Another potential application of CLE could be repeated screening for tumor free resection margins during surgery. Here, topical application of dyes and superficial imaging may suffice to differentiate healthy brain from residual tumor tissue. Increase of the surgical resection in malignant brain tumors improves the clinical outcome and life time of those patients.

For a future workflow, we additionally plan to integrate new optical imaging techniques, such as *in vivo* CLE, into robotic-assisted/−guided surgical systems. Therefore, confocal endomicroscopic probes need to be miniaturized even further, such as done with probes that can be inserted into the working channel of conventional colonoscopes used in recent studies [36], [37]. Computerized algorithms, similar to those recently described [12], might be used for automated and accurate tumor detection and diagnosis. Miniaturized robotic arm holding systems, remotely operated by the physician, will then perform minimally invasive tumor resection and/or chemo-application in the same surgical session.

In summary, our study shows that *in vivo* and *ex vivo* CLE is possible in a rodent model of malignant glioma and human tissue using confocal laser endomicroscopy. Histomorphologic discrimination between neoplastic and healthy tissue based on characteristic fluorescent staining patterns and unique morphologic features was achieved *in vivo* and *ex vivo*. Also, intravital functional and molecular imaging seems to be within reach. These findings might have impact on our future clinical approach to intracranial neoplasia and other forms of cancer. For neurosurgical use the goals which can be achieved with this system are 1) an immediate intraoperative histopathological differentiation of brain tumors, 2) the more accurate extended surgical resection of infiltrating malignant brain tumors, and 3) a more precise resection of skull base tumors without damage to important brain structures such as nerves and vessels because of a better optical differentiation between tumor, nerve, vessel and dura mater at the cellular level.

## Supporting Information

Figure S1 **Detail of the confocal endomicroscopy probe. A–D** Dimensions and handling of the confocal probe. The diameter of the tip of the probe is 5 mm with a shaft diameter of 7 mm. The shafts length is 150 mm, but systems with a longer probe are also available commercially.
(TIF)

Figure S2 **Additional images of different human brain tumor biopsy specimens.** A, B - Histopathologic (left) and confocal laser endomicroscopic images of a craniopharyngeoma biopsy specimen. A close resemblance in nuclear patterns can be observed. C, D – Histopathologic (left) and confocal laser endomicroscopic (right) images of a breast cancer metastasis biopsy specimen. Large cells with prominent nuclei can be visualized.
(TIF)

Figure S3 **Additional images of different human brain tumor biopsy specimen.** A, B - Histopathologic (left) and confocal laser endomicroscopic images (right) of a meningeoma biopsy specimen. C, D – Histopathologic (left) and confocal laser endomicroscopic (right) images of a epidermoid tumor biopsy specimen.
(TIF)
