# Supplementary figures and images for: Confocal Laser Endomicroscopy for Diagnosis and Histomorphologic Imaging of Brain Tumors In Vivo
Source: PLoS One. 2012 Jul 24;7(7):e41760. doi: 10.1371/journal.pone.0041760 (PMC3404071; doi:10.1371/journal.pone.0041760)

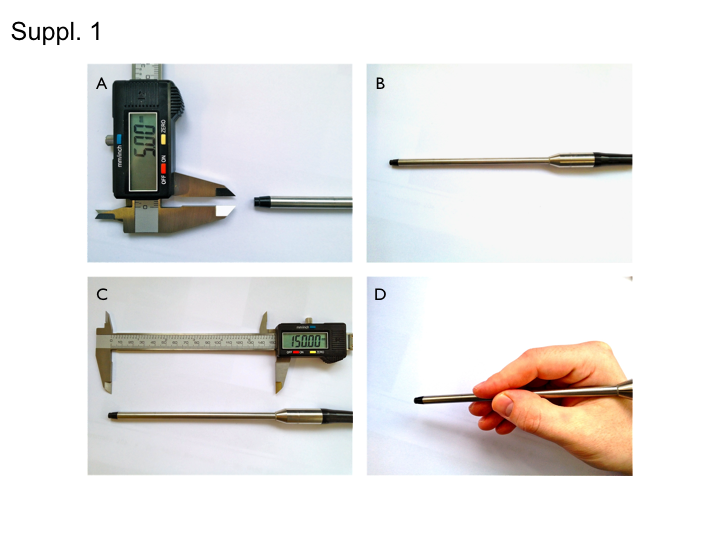

Supplement: Figure S1 — Detail of the confocal endomicroscopy probe. A–D Dimensions and handling of the confocal probe. The diameter of the tip of the probe is 5 mm with a shaft diameter of 7 mm. The shafts length is 150 mm, but systems with a longer probe are also available commercially. (TIF) [file pone.0041760.s001.tif]

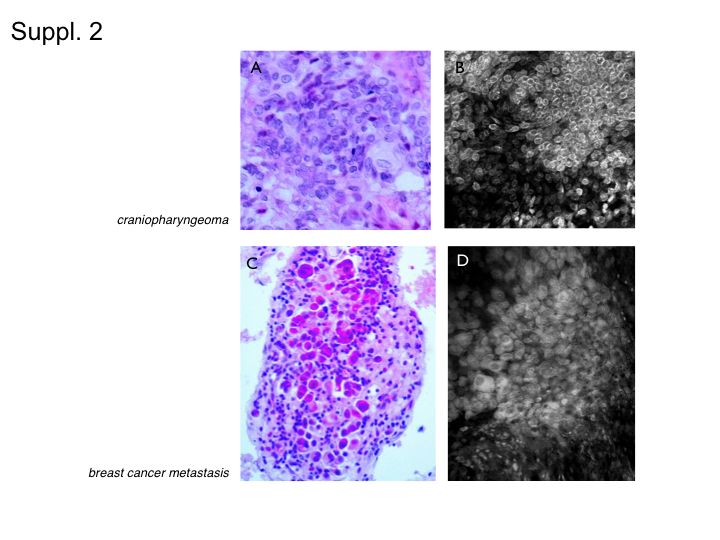

Supplement: Figure S2 — Additional images of different human brain tumor biopsy specimens. A, B - Histopathologic (left) and confocal laser endomicroscopic images of a craniopharyngeoma biopsy specimen. A close resemblance in nuclear patterns can be observed. C, D – Histopathologic (left) and confocal laser endomicroscopic (right) images of a breast cancer metastasis biopsy specimen. Large cells with prominent nuclei can be visualized. (TIF) [file pone.0041760.s002.tif]

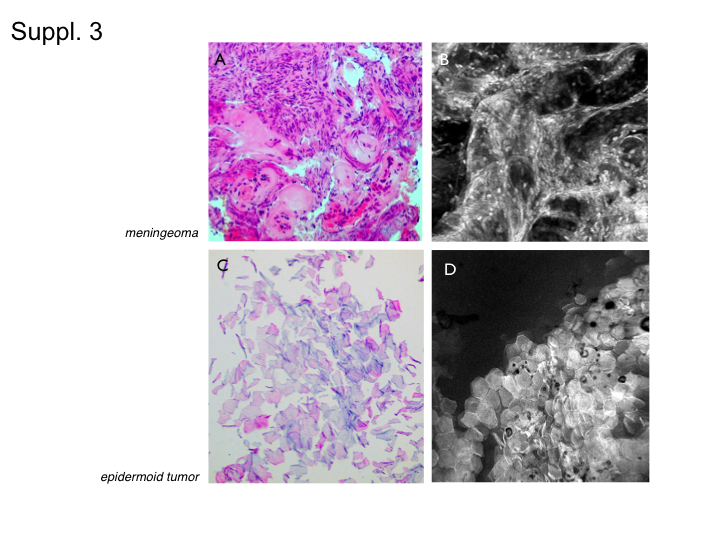

Supplement: Figure S3 — Additional images of different human brain tumor biopsy specimen. A, B - Histopathologic (left) and confocal laser endomicroscopic images (right) of a meningeoma biopsy specimen. C, D – Histopathologic (left) and confocal laser endomicroscopic (right) images of a epidermoid tumor biopsy specimen. (TIF) [file pone.0041760.s003.tif]
